# Consensus of the 'Malasars' traditional aboriginal knowledge of medicinal plants in the Velliangiri holy hills, India

**DOI:** 10.1186/1746-4269-4-8

**Published:** 2008-03-27

**Authors:** Subramanyam Ragupathy, Newmaster G Steven, Murugesan Maruthakkutti, Balasubramaniam Velusamy, Muneer M Ul-Huda

**Affiliations:** 1 Floristic Diversity Research Group, OAC Herbarium, University of Guelph, Guelph, Ontario, N1G 2W1, Canada; 2 P.G. and Research Department of Botany, Kongunad Arts and Science College, Bharathiar University, Coimbatore, Tamil Nadu, India

## Abstract

There are many vanishing cultures that possess a wealth of knowledge on the medicinal utility of plants. The Malasars of Dravidian Tamils are an indigenous society occupying the forests of the Western Ghats, South India. They are known to be exceptional healers and keepers of traditional aboriginal knowledge (TAK) of the flora in the Velliangiri holy hills. In fact, their expertise is well known throughout India as evidenced by the thousands of pilgrims that go to the Velliangiri holy hills for healing every year. Our research is the first detailed study of medicinal plants in India that considers variation in TAK among informants using a quantitative consensus analysis. A total of 95 species belonging to 50 families were identified for medicinal and general health purposes. For each species the botanical name, family, local name, parts used, summary of mode of preparation, administration and curing are provided. The consensus analysis revealed a high level of agreement among the informants usage of a particular plant at a local scale. The average consensus index value of an informant was F_IC _> 0.71, and over 0.80 for some ailments such as respiratory and jaundice. Some of the more common problems faced by the Malasars were gastrointestinal disorders, respiratory illness, dermatological problems and simple illness such as fever, cough, cold, wounds and bites from poisonous animals. We also discovered several new ethnotaxa that have considerable medicinal utility. This study supports claims that the Malasars possess a rich TAK of medicinal plants and that many aboriginals and mainstream people (pilgrims) utilize medicinal plants of the Velliangiri holy hills. Unfortunately, the younger generation of Malasars are not embracing TAK as they tend to migrate towards lucrative jobs in more developed urban areas. Our research sheds some light on a traditional culture that believes that a healthy lifestyle is founded on a healthy environment and we suggest that TAK such as that of the Malasars may serve toward a global lifestyle of health and environmental sustainability.

## Background

There are many vanishing cultures that possess a wealth of knowledge on the utilization and conservation of plants. Much of the traditional aboriginal knowledge (TAK) [1-3] concerning new drugs was discovered before the middle of the last century [4], but has risen again in the last decade [5]. New fields have developed such as the "economics of identity", which bridge the economics of aboriginal and scientific classification [6]. The recent interest in this area of research is partly driven by society's interest in healthy lifestyles, which supports a rapidly growing $230 billion dollar market force in USA alone [7]. The World Health Organization [8] estimates that 80% of the world's population relies on traditional healing modalities and herbs. Many cultures still maintain traditional medical systems based on TAK and researchers are exploring cultural health and success based on TAK [7,9-11]. These traditional cultures believe that a healthy lifestyle is founded on a healthy environment and some recent research on local or traditional ecological knowledge (LEK or TEK) has improved natural resource conservation and management policies for modern society [3,12-16].

India is rich in its ethnic diversity of which many aboriginal cultures have retained traditional knowledge concerning the medicinal utility of the native flora. Southeast Indians have been known to put a great emphasis on traditional knowledge systems and practices, which is supported by their vast intra-ethnic diversity [17]. India has over 537 different aboriginal and other ethnic groups constituting approximately eight percent of the country's population [18,19]. Traditional knowledge systems including various medicinal plant utilities appear to vary according to local population domain [20]. Documentation of these local knowledge systems concerning medicinal plants may have high impacts from a bioeconomic point of view [6]. Tribal communities living in biodiversity rich areas possess a wealth of knowledge on the local utilization and conservation of food and medicinal plants [18,21]. This traditional knowledge, which developed over years of observation, trial and error, inference and inheritance, has largely remained with the aboriginal people [22,23]. However, these cultures and their associated botanical knowledge may be in peril and may even become extinct. Migration from one area to another in search of improved livelihoods is a key feature of human history. Many aboriginals in India migrate to access emerging opportunities and industrialization. This widens the gap between TAK and modern knowledge associated with workplace and social skills of the developed mainstream populations. It is a fact that as traditional healers who value TAK are becoming very old; younger generations exhibit a lack of interest in TAK with a trend toward migration to cities for lucrative jobs. TAK in India is declining [24,25].

The study of ethnobotanical research is deeply rooted within India. There are many examples of medicinal ethnobotanical surveys conducted in India in the past that have recorded many botanical remedies among many aboriginal groups: Malasars [26]; Malamalasars [27]; Malayalis [28-31]; Irulas [22,23,32-34]; Gonds [15]; Koysd, Konda reddis, Valmikis, Koyas, Chenchus, Lambadis, Jatapus, Savaras, Bagatas, Kammaras, Khondas, Nukadoras, Porjas, Jatapus [35]; Paliyar [36]; Kanikar [37]; Todas, Kotas [38,39]; Kattunayakas [40]; Apatani [41]; Chellipale [42]. Although there are many descriptive qualitative surveys of TAK, to our knowledge, there are no ethnobotanical studies within India that consider variation in TAK among informants using a quantitative consensus analysis.

Aboriginal knowledge about plants needs to be reliable and repeatable if it is used as a bridge in scientific inquiry with an application to medicine and society-at-large. Trotter and Logan [43] presented a quantitative method to evaluate consensus among informants in order to identifying potentially effective medicinal plants. In the last 20 years since Trotter and Logan's [43] publication there has been limited research from several countries: Peru [44]; Indonesian Borneo & Timor [45,46]; Northeastern Brazil [47,48]; Mexico [5,9,49]; Chile, Colombia, Ecuador, Guatemala [50]; Southern Belize [51]; Kenya [52,53]; Mali [54]; Ethiopia [55]; Tanzania [56,57] and the Canadian Arctic [58]. This body of literature suggest that there is considerable variation in consensus factors and how this technique has been implemented. Moerman [59], Phillips and Gentry [44] and Heinrich [5] readapted Trotter and Logan's [43] factor of informant consensus factor (F_IC_) in order to quantitatively evaluate the degree of selection of certain plants for a particular utility (e.g., ailment). One of the traditional intentions of F_IC _is to test the homogeneity among informants' knowledge [43]. In fact some researchers use consensus analysis to test falsifiable hypotheses concerning informant selection and use of plants [53,44]. Many other researchers have employed consensus analysis as a decision making factor [5,48] to examine the variation in TAK of cultivars by traditional aboriginal farmers [49], weighing the relative importance of TAK [60], identifying discrepancies in ratings [50], estimating the competence of informants [61,62,50] and ethnopharmacolgical surveys [54,48,47,55].

The theoretical importance of our study is to test consensus (reliability/repeatability) of TAK within one ancient culture; the Malasars of the Velliangiri hills in the Western Ghats of Nilgiri Biosphere Reserve, India. We chose to work with the Malasars of India, because 1) there are known to be exceptional healers and keepers of TAK of the flora in the Velliangiri holy hills [63] and 2) there is limited research on the Malasars TAK [64]. We hypothesize that consensus of TAK of specific plants used for different illness categories are high indicating reliable and repeatable TAK among informants at a local scale (within one localized aboriginal group – Malasars of the Velliangiri hills), because it has been used within their culture without interruption for many generations. Scientific inquiry demands repeatability in order to substantiate claims of medicinal utility within any aboriginal culture. Alternatively, consensus of TAK may be low at local scales [51] because of i) unreliable TAK, ii) informant bias, iii) local remedies; certain villages may have unique uses for plants, iv) variability in local ethnotaxa; certain communities may have found variants or ecotypes for some plants that result in unique qualities that are of particular use at only a local scale, iv) use of pharmaceutical supplements; the availability of modern pharmaceuticals for a particular ailment may result sporadic use of traditional remedies and v) availability of multiple remedies; there may be groups of plants and therefore several remedies available that are preferentially selected by individual healers for various utility (e.g., healing some ailment), thus indicating the potential biological activity for a group of plants [59]. These groups may represent Linnaean taxa (i.e., genus or family) that share similar biological processes, or aboriginal classifications may group plants (e.g., 'chedi' or 'kodi' etc.) that serve a similar utility [65,25].

## The Malasars and their land

### Ethnography

**Figure 1:**
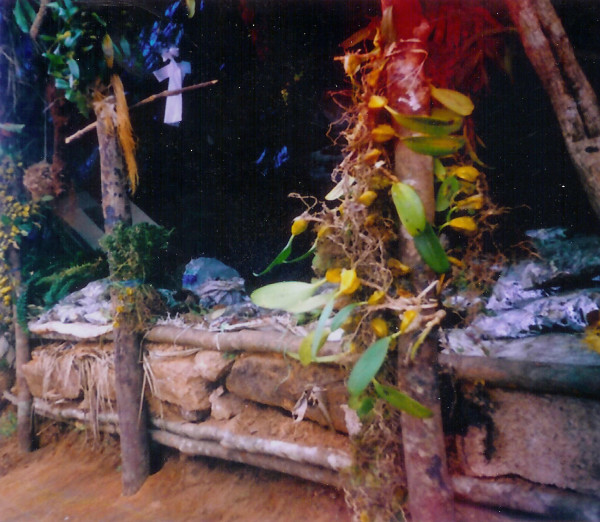
Malasars medicinal plant market place.

Murugesan [63] and Murugesan et al [66] previously described ecosystems and aboriginal communities for our area of study. The Malasars (etymology in tamil – mala = hill; saras = people who live in and depend on the hills) are an aboriginal community who reside in the forest of the Velliangiri holy hills. They are traditionally hunter gathers. In the Velliangiri hills their settlements were situated near Poondi. The Malasars are considered the 'lords of the hills'. Luiz [67] and Jakka [68] stated that there is no information regarding the origin and early history of the Malasars. They appear to be an original aboriginal group of the hills in the earliest of records. The isolation provided by the hills and inaccessible forests preserved some forms of old dialects of the Dravidian language family (*Official records of the Directorate of Tribal Development, Tamil Nadu*). The Malasars are restricted only to Tamil Nadu and the adjacent State of Kerala. The Malasars are considered to be part of the Dravidian family, which are known to speak Telugu, Malyalam and Tamil in South India, of which the 'Malasars' speak only Tamil and Malayalam. Their lifestyle and dialogs are influenced by surrounding habitats (mountain) and the mainstream people who make a pilgrimage to the hills. They reside in hamlets known as 'pathis' formed of huts made of thatched bamboo and plastered with mud. The Malasars have their own deities, some of which are 'Mallung', 'Kali' and 'Mariamman'. 'Mallung' is represented by a stone encircled by a wall, serving as a temple, where goats and cocks are offered as sacrifice. The Malasars believe that neglectful respect to the 'Mallung' can lead to the death and injury of people by attacks from tigers/elephants/wild buffalo. They are non-vegetarians and eating the meat of all categories of wild birds and animals. They have accumulated extensive knowledge of plant and animal utility, which may be attributed to their long association with the rich flora of the hills and their socioeconomic system, which relies mainly on non-timber forest products such as the harvest of native fruits, turmeric, ginger and honey (Figure 1). Farming is not a common occupation and is limited and primitive. More recently, many young 'Malasars' work as 'coolies' in the forest operations, employed as agricultural labourers.

### Biogeography

**Figure 2:**
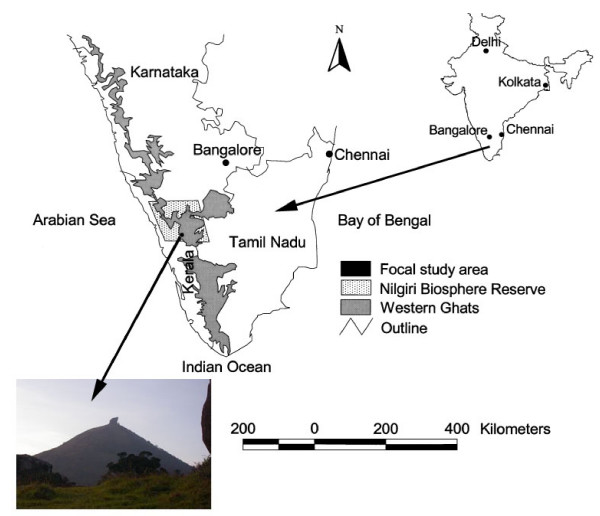
Location of the study site in the Velliangiri hills located on the Nilgiri Biosphere Reserve, Westen Ghats, India (Map modified from Kodandapani et al [69]).

**Figure 3:**
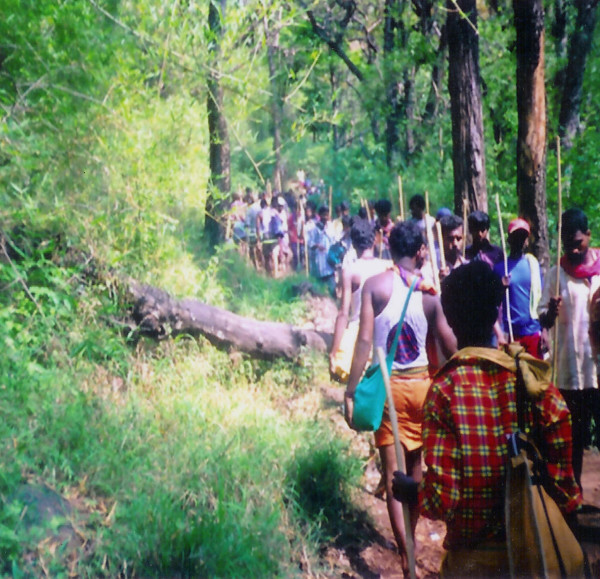
Pilgrims walking with bamboo poles through jungles.

The Velliangiri hills forms a major range in the Western Ghats that is rich in biodiversity and largely untouched by development because of its cultural and religious importance (Figure 2) [69]. It is popularly known as "Thenkailaya malai" (in Tamil), the holy hills of southern India; "Kailaya malai", which is located close to the Himalayas, is the holy hill of northern India. The Velliangiri Andavar temple and the cave of "Panchalingas" are popular pilgrimages within the Velliangiri hills. Hundreds of pilgrims visit the Velliangiri Andavar temple bare foot every new moon. It is a dangerous trek through grasslands and forests with wild bisons, elephants and poisonous snakes. This concludes with a 10 km hike up the steep hillside through a tropical moist deciduous forest with many thorny shrubs. Devotees hike bare foot as they believe that animals will attack them if they wear shoes. The pilgrims start walking up the hill early in the morning and climb down before dark (Figure 3). We participated in a floristic investigation that revealed considerable diversity (1715 species of angiosperms including 439 endemics) within the Velliangiri holy hills [63,66]. It is astonishing that this relatively small (48 sq. km.) holy reserve contains over half of the angiosperm diversity as defined during comprehensive surveys of the large (5520 sq.km.) Nilgiri Biosphere Reserve [70-75,63]. Field biologists must adhere and respect these religious customs, which places a restriction on how far they can explore in one day. This may explain why botanists have not fully explored the richness of this unique flora. In fact there are only a few historical botanical collections from the Velliangiri hills made by Raju and Rathinavelu (in 1932), Sebastine (in 1959), Vajravelu (in 1972) and Chandrabose and Karthikeyan (in 1978). During our recent floristic surveys (2003 – 2007) of the Velliangiri hills we increased these collections considerably and discovered several new species to science while working with the knowledgeable elders of several local aboriginal communities [66].

The study site (longitude 6° 40' to 7° 10' E and latitude 10° 55' to 11° 10' N) is located within the Velliangiri holy hills, which forms a major range in the Western Ghats in the Nilgiri Biosphere Reserve. The research was conducted among seven hills with altitudes ranging from 520 m – 1840 m, which is bordered by the Palghat district of Kerala on the western boundary, the plains of Coimbatore district to the east, the Nilgiri mountains to the north, and the Siruvani hills on the southern boundary (Figure 2). The annual rainfall is quite variable in the hills (500 mm – 7000 mm) with temperatures ranging from 0°C during winter to 41°C in the summer. Many seasonal rivers such as the Neelivaikal, Mayar or Andisunai traverse the hilly landscape. The "Noyyal" river originating from Velliangiri hills is one of the major tributaries of the Cauvery, which irrigates about 100,000 of hectares of agricultural land in the plains. The Velliangiri hills watershed feeds into the Siruvani dam, which is the only drinking water for 150,000 people in the urban centre of Coimbatore.

## Methods

### Ethnobotanical survey and consensus analysis

The interview protocols, data confirmation and field observation were all followed as suggested by Bernard [76]; Etkin [77]; Pelto and Pelto [78]; Alexiades [79]. To elucidate community domains and determine differences in knowledge among the 'Malasars' people, we cross checked with other Malasars respondents. With the help of the headman, we were able to record information on the local customs, habits and beliefs, information on the surrounding area and individuals who are knowledgeable of the local flora [80].

Surveys of informant TAK of medicinal plants were used for the consensus analysis. Local traditional healers having practical knowledge of plant medicinal utility of the Velliangiri hills were interviewed during April 2003 – January 2007. During the course of the study, about 18 field trips were conducted in the study area totalling 120 days. Surveys were conducted by a stratified random selection of informants, based on methods suggested by Schultes [81,4], Jain [18] and Bernard [76].

**Figure 4:**
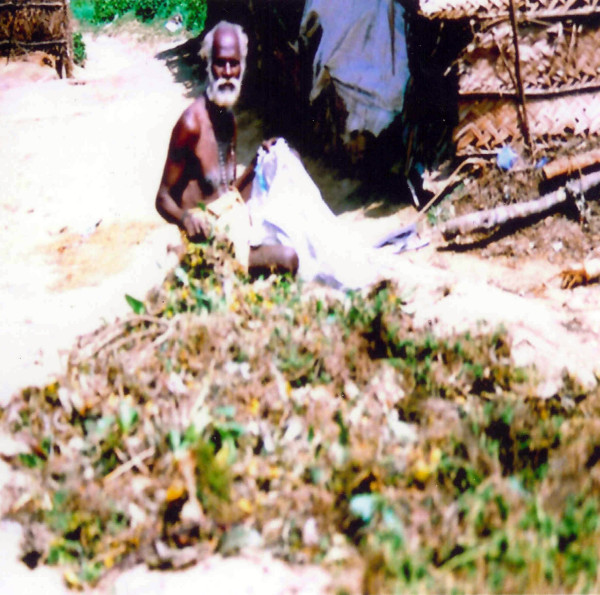
Aboriginal elder sun drying and selling medicinal bulbs.

Successive free listing was used to interview 80 knowledgeable informants providing data for the consensus analysis. Knowledgeable informants were selected following standard interview protocols [76,82,83], which included verification whether these informants were traditional healers within their communities (Figure 4). We interviewed over 120 informants of which we chose 80 knowledgeable informants equally distributed among four different age categories; elders, middle aged, teenagers, <10 years. During this interview we documented all possible information about a specific plant and then sorted this information according to utilitarian perspective, local name or ecology. The informants were given limited time and there was no differentiation among gender. We also asked the informants to group the plant specimens into different illness categories. We requested all informants to collect specimens of the plants they knew or to show the plant species on site. Interviews were conducted in the regional language, Tamil. The questionnaires were used to obtain information on medicinal plants with their local names, parts used, mode of preparation and administration.

Calculation of a consensus factor (F_IC_) for testing homogeneity on the informant's knowledge was followed by the method provided by Trotter and Logan [43]. A consensus factor of F_IC _is given by:

F_IC _= N_ur_-N_t_/(N_ur_-1)

The factor provides a range of 0 to 1, where a high value acts as a good indicator for a high rate of informant consensus. N_ur _is the number of use-reports of informants for particular illness usage, where a use-report is a single record for use of a plant mentioned by an individual, and N_t _refers to the number of specie used for a particular illness category for all informants. The majority of illness types are grouped into predefined ethno/economic botany categories [84,5], with the additions of a few other illness categories (Table 1), which were commonly mentioned during our interviews because they were prevalent in these communities. The use of "general categories" is adopted here as recommended by other ethnobotanical researchers [84,5]. These 51 illnesses were sorted into 10 usage categories (Table 1). All of the illness types were translated as best as possible from the Malasars description of the illness/symptoms to known biomedical/english terms, with few exceptions (e.g., spiritualism – repel evil).

**Table 1:** 51 Malasars ailments grouped by Illness category [84].

**Illness category [84]**	**Biomedical Term**	**Malasars Term**
Dermatological	*Blisters*	Koppalam
	*Dandruff*	Podugu
	*Eczema*	Akkii
	*Heel cracks*	Padha vedippu
	*Leucorrhœa*	Vellai paduthal
	*Luecoderma*	Ven theymbal
	*Piles*	Moolam
	*Psoriasis*	Sori
	*Skin allergies*	Sirangu
	*Skin disease*	Thol viyathi
Fever	*Fever*	Kaichal
Gastrointestinal	*Diarrhoea*	Vaitru po'kku
	*Dysentery*	Seetha baeathi
	*Dyspesia*	Vairty kadupu
	*Gas trouble*	Vaivu
	*Intestinal worms*	Vaitru poochi
	*Purgative*	Vaitru po'kku
	*Stomach ache*	Vaitru vali
	*Stomach ulcers*	Vaitru pun
General health	*Antidotes*	Visha murichi
	*Blood circulation*	Ratha o'ttam
	*Blood pressure*	Ratha ashutham
	*Body wash*	Udampu podi
	*Cold*	Shzali
	*Giddiness*	Mayakam
	*Hair dye*	Mudi sayiam
	*Heart disease*	Irudhaya no'i
	*Hallucinogenic*	Beedi
	*Mental disorders*	Moolai ko'laru
	*Night blindness*	Kamalai
	*Paralisis*	Vadham
	*Power of memory*	Ganbaga sakthi
	*Tooth Caries*	Pal poochi
	*Tooth cavities*	Pal sothai
	*Vitamins*	Kayakalpa
Infections	*Antiseptic*	Ethir nachu
	*Wounds*	Kayam
Pain	*Body pain*	Udambu vali
	*Ear ache*	Kadhu vali
	*Epilepsy*	Kaka valipu
	*Headache*	Thalai vali
	*Muscular pains*	Sathai pidipu
	*Rheumatic pain*	Moottu vadham
	*Stomach ache*	Vaitru vali
	*Tooth ache*	Pal vali
Respiratory	*Bronchitis*	Ma'r sali
	*Cough*	Irummal
Evil spirit	*Repel evil*	Peay viratti
Urinary	*Diuretic*	Neer vadithal
	*Urinary tracts*	Kuzhai adaipu
*Jaundice*	*Jaundice*	Manjal kamalai

### Botanical documentation and preservation

The identity of spontaneously described plants found in the Velliangiri hills was confirmed by reference to fresh plant material collected, and to voucher specimens of known identity [85,86]. The Linnaean identities were designated by comparing the specimens with the authentic type specimens in herbaria, and by referring to recent taxonomic monographs and revisions. The botanical nomenclature followed that of the Flora of Tamil Nadu, India Series Analysis [87-89]. They were verified at Botanical Survey of India, Southern Circle, Coimbatore, India. All the preserved herbarium voucher specimens are deposited in the herbarium of Kongunad Arts and Science College (KASC) and herbarium of Botanical Survey of India, Southern Circle (MH).

## Results and discussion

### Diversity in Malasars TAK of medicinal plants

**Figure 5:**
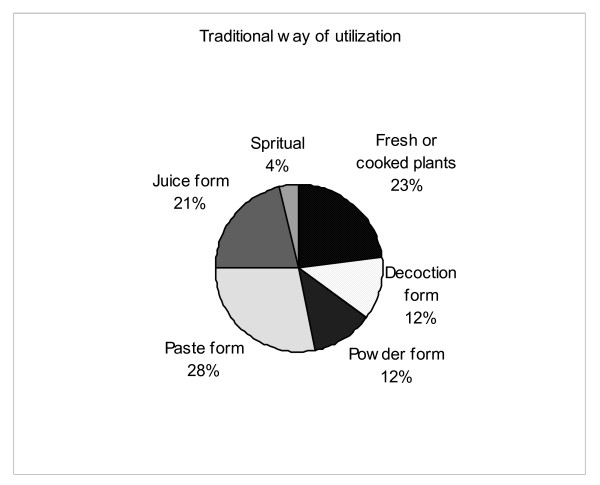
Categories of Malasars mode of utilization for various ailments.

The Malasars preferred to use a diversity of native plants with medicinal utility. A total of 95 species distributed in 85 genera belonging to 50 families were identified for medicinal and general health care purposes during this study (Additional file 1). For each species we provide the Linnaean taxonomy, ethnotaxonomy, preparation method and medicinal use (Additional file 1). TAK concerning medicinal utility in our study supports much of the TAK in Pandi Kumar's [26] study of illness, remedies and mode of action. However, there are few disagreements among remedies in this study and that of Pandi Kumar's study [26]. For example, Pandi Kumar [26] noted that *Datura metal *L. is used to heal wounds; in our study it is used to cure cold and body aches. There is no consensus analysis in Pandi Kumar's study with which to evaluate the reliability of the informants TAK and make comparisons with the consensus of the respective TAK in our study. The Malasars prefer to utilize species from primary or secondary semi-evergreen rainforests of Velliangiri hills, rather than the weedy species from disturbed areas. The most common families in the study were Euphorbiaceae (6 species), Fabaceae (6 species) Acanthaceae (5 species), Boraginaceae (4 species), Cucurbitace (4 species) and Rutaceae (4 species) (see complete list of families in Additional file 1). Herbs (43 species) were the most common functional group of plants followed by climbers (18 species), trees (18 species) shrubs and (16 species). The Malasars healers use many different plants for the same ailment and some plants can be used for different ailments. For example, Malasars healers commonly use many plants to treat wounds, cold, cough, fever, body pain; These include, *Achyranthes aspera *Blume, *Acorus calamus *Linn., *Amaranthus spinosus *L., *Azima tetracantha *Lam., *Blepharis repens *(Vahl) Roth, *Cinnamomum macrocarpum *L., *Datura metel *L., *Leucas aspera *(Willd.) L., *Malaxis rheedii *Sw., *Mollugo nudicaulis *Lam. and *Terminalia chebula *Retz. As mentioned earlier, for a single illness there can be many plants used to cure it, resulting in a low consensus factor. A Malasars healer could treat a general cough with either *Acorus calamus *Linn., *Cinnamomum macrocarpum *L., *Piper longum *Miq. and *Terminalia chebula *Retz. The preference for use may be related to availability, cost or possible interactions with other plants currently being taken by the patient. An assortment plant parts were utilized as medicine by the Malasars of which the leaves were used most frequently, followed by roots, bark, seeds, whole plants, flowers, fruits and latex/sap. The preparation for utilization of these plant parts can be grouped into several categories with those for ingestion most commonly utilized; freshly cooked, paste and juice preparations (Figure 5).

The Malasars demonstrated diverse medicinal utility of the local plant flora in the Velliangiri hills. Our interviews yielded ten illness categories including 51 medical uses (Table 1). These illness categories were modified according to Cook [84] and Heinrich [5]. Diversity in medicinal utility may be attributed to a diversity of ailments within the Malasars or neighbouring communities. However, we did not study medical ailments of the Malasars or the neighbouring communities in great detail and are not aware of any published research on this topic. Treating illnesses with particular plants, such as piles (*Tinosporia cordifolia *(Willd.) Hook. f. & Thoms. *Zizyphus maurtiana *Lam.), stones in urinary tracts (*Boerhavia erecta *L.), leucoderma (*Kalanchoe floribunda *W. & A.), leucorrhoea (*Kalanchoe floribunda *W. & A., *Securinega leucopyrus *(Willd.) Muell., *Dichrostachys cinerea *(L.) W. & A., *Plumbago zeylanica *L., *Centella asiatica *(L.) Urban), epilepsy (*Indigofera caerulea *Roxb.), eczema (*Centella asiatica *(L.) Urban, *Glinus lotoides *L.) by Malasars healers is unique and may be indicative of the need to treat more frequent ailments within their community. Poisonous bites are also a frequent treated because the Malasars work in the fields and forests where snakes and scorpions are commonly encountered. Perhaps a more likely explanation for the high diversity of medicinal utility is that the Malasars are known to be great herbal healers and treat many people from urbanized, mainstream communities. In fact, many Malasars earn their living treating people's ailments using the flora of the Velliangiri hills.

### Consensus of TAK among Malasars informants

Our research indicates a high level of consensus within the Malasars community. This is the first consensus analysis research published from an aboriginal group in India and supports many reports of the rich botanical knowledge of aboriginals within India [64]. Sajem and Gosai [90] reported consensus of medicinal use of plants in northeast India. They did not use the quantitative method proposed by Trotter and Logan's [43], but rather defined consensus as the percentage of informants who listed a particular utility for a specific plant. In our study, the informant consensus of medicinal plant usage with the Malasars resulted in F_IC _factors ranging from 0.5 to 0.92 per illness category (Table 2). The average F_IC _value for all illness categories was 0.71, indicating a significant level of informant consensus compared to similar studies from other countries [5,51]. In the literature, high informant consensus (F_IC _0.875) was also recorded among the snakebite healers of Kamba in Africa [52], treating 'mich' or febrile diseases (F_IC _0.80) [55], and respiratory disorders (F_IC _1.00) among Inuit in Nunavut [58]. A high consensus factor may indicate that there is some key phytochemical ingredient(s) in these plants which requires phytopharmacological analyses. Our research revealed that the category jaundice included only 2 species (N_t_), resulting in a high F_IC _factor of 0.92, indicating greater homogeneity among informants. Although this illness category was not used by Cook [84] in his study, it is an integral part of the Malasars medicinal concepts. This particular illness is sporadic though out India and is cured effectively by *Phyllanthus amarus*, a botanical remedy that is known to aid the liver – Ayurvedic medicine for jaundice [91-93]. However, Malasars informants consistently reported the use of *Euphorbia thymifolia *L. and *Indigofera caerulea *Roxb. to treat jaundice. The 'Malasars' have also identified subspecies or ethnotaxa for treating jaundice. These ethnotaxa are morphologically similar to *Phyllanthus amarus *but differ in habitat and/or other taxonomic characters that are unique to their classification system. The identity of ethnotaxa is not unique in India as we have documented many ethnotaxa used by the Irulas in Tamil Nadu [65,25].

**Table 2:** Ethnobotanical consensus index for traditional medicinal plant use categories.

**Illness category [84]**	**Number of Taxa (N_t_)**	**Number of use-reports (N_ur_)**	**Informants' consensus index factor (F_ic_)^a^**
Jaundice	2	14	0.92
Fever	3	5	0.50
Repel evil sprit	5	31	0.87
Respiratory	6	27	0.80
Infections	6	14	0.61
Dermatological	14	51	0.74
Pain	16	65	0.76
Gastrointestinal	18	41	0.56
General health	25	86	0.71
Wounds	6	15	0.62
**Total^b^**	101	249	-

^**a**^F_ic _= N_ur_-N_t_/(N_ur_-1), providing a value between 0 and 1, where high value indicates a high rate of informant consensus.

^**b**^A taxon may be listed in several of the categories of medicinal usage.

There may be a logical explanation for some of the lower consensus factors in our study. The low consensus factor (F_IC _0.56) for the gastrointestinal category may be indicative of the lack of gastrointestinal disorders among the Malasars. Studies of other cultures have shown high incidents of gastrointestinal occurrences, but among the Malasars it is relatively low [24,94,95]. While the actual reason for this is unclear, the ratio of use-reports to number of taxa might be a reason for this [51,43,44]. We currently are investigating the incidence of gastrointestinal disorders in other aboriginal communities (eg. Muthuvans, Irulas) within the same geographic area. The low consensus factor (F_IC _of 0.50) for the fever illness category may be explained by several factors. The availability of easily accessible pharmaceuticals provides many alternatives to traditional medicine. This may reduce the use of some traditional remedies, which could reduce consensus of TAK for some common ailments such as fever. For example, many of the local shops sell cheap allopathic/pharmacological medicine that provides quick relief for fever reducing the need for traditional fever remedies. An alternative explanation for a low consensus factor may be that there are a variety of plants being used for a variety of fever causations, such as sore throat, cold and flu.

Our consensus research provided new insights for several other categories of medicinal utility by the Malasars of which we learned that they routinely consume plants for their vital well being and good health. The "general health" category is not included in the standardized illness groupings by Cook [84]. We included this category because it is an integral part of the Malasars health concept of which healers insist on having plants as part of their diet to maintain good health. The general health category included the largest number of taxa, reports of utility and a relatively high level of consensus (Table 2). We found in our survey that some of the plants used in the general health category are edible to the Malasars (7 species), while others were non-edible (18 species) (Additional file 1). An ancient tradition of the Malasars is to eat certain plants on a regular basis according to the seasons in order to prevent certain diseases. It is common practice for the Malasars to consume plants that they come across while out on walks, collecting water or any other daily routine. They believe it will aid their general health and provide an ailment for chronic disorders; examples include blood circulation (*Begonia malabarica *Lam), diuretic (*Coccinea grandis *(L.) J. Voigt), and bronchitis (*Mukia maderaspatana *(L.) M. Roem).

**Figure 6:**
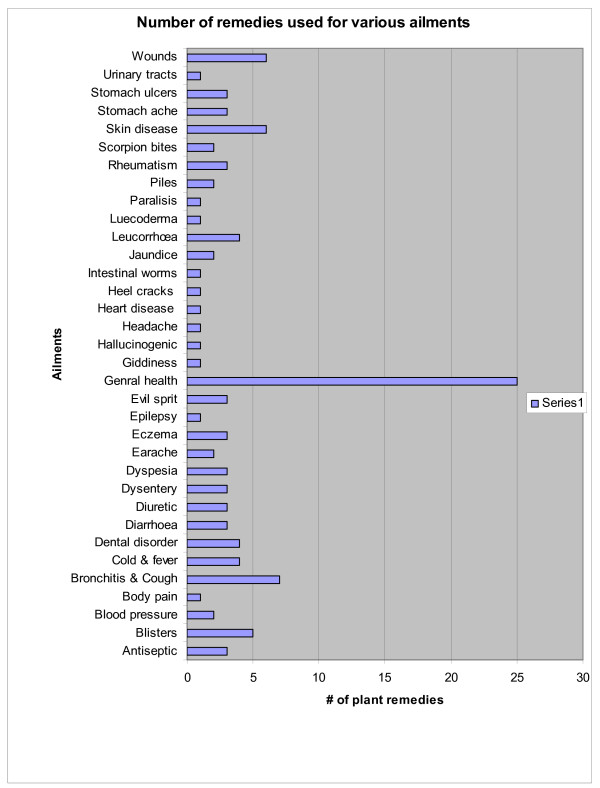
Number of plants used for various illnesses.

Consensus analysis is a crucial tool in establishing a comparative estimation of the level of informant consensus on the use of medicinal plant remedies [52]. We found the consensus analysis a useful tool to confidently reveal 95 species used by the Malasars to treat 51 ailments (Table 1; Figure 6). Leaman et al [45] underscored the use of consensus analyses in the discovery of 17 traditional plant remedies used by the Apo Kenyan to treat malarial infection. More recently, Kisangau et al [56] used this method when studying the Haya aboriginals in Tanzania, which revealed 75 plant remedies (F_IC _0.70) used to treat HIV/AIDS. Schlage et al [57] used the consensus analyses to identify the importance Washambaa TAK in the daily treatment of many ailments in Tanzania. Although consensus analysis is a great tool, we agree with other researchers [48,51,9,49,47,5,56,57,55] that there are some factors that limit the power/reliability of a consensus analysis, namely a) low numbers of knowledgeable informants within a local culture, b) heterogeneous use, and c) low numbers of surveys.

### Malasars TAK

The Malasars have unique medicine in relation to other aboriginal groups in India. Several ethnobotanical studies have enumerated the plants used for various illnesses in India and elsewhere, especially wound healing [22-24,96] and skin diseases [97,98]. However when we compared these reports to the Malasars healers we found that they occasionally use different medicinal plants for the same illness category. To heal wounds, the Malasars used six plants (*Achyranthes aspera *Blume, *Azima tetracantha *Lam., *Blepharis repens *(Vahl) Roth, *Euphorbia hirta *L., *Malaxis rheedii *Sw., *Trichodesma indicum *(L.) R. Br.), of which all but one (*Malaxis rheedii *– high altitude species) are distributed in the plains and coastal areas. In this case personal preference may not be the reason for choosing these plants, but potential active ingredients for utilization in these plants for particular illnesses. Similarly, for skin diseases, three plants are used (*Acalypha indica *L., *Lycopodium phlegmaria *L. and *Sphaeranthus indicus *L.) of which all but *Lycopodium *are commonly found in the plains. In this case preference of availability may not be the key reason for constant utilization of this plant by the Malasars for skin diseases. However, on the plains, *Acalypha indica *is also used by traditional healers to treat scorpion bites and sore throats [22,23,30].

The Malasars are strong believers in spiritualism. They have a special way of dealing with illnesses brought along by evil. This is known as a culture bound syndrome, a folk illness that is specific to different cultures [99]. This is similar to '*susto*', within the latin population of Mexico, Guatemala and Texas. *Susto *is a folk illness, specifically a "fright sickness" with strong psychological overtones. The Malasars cure this category of illness using plants such as *Abrus precatorius *L., *Crotalaria verrucosa and Selaginella rupestris *(see mode of utilization in Additional file 1). The informant consensus factor for the spiritual illness category was quite high (F_IC _of 0.87). Weller et al [99] also reported that the treatment for *susto *involves praying for the individual, discussing the event that brought about the "sickness" and cultural rituals that involve 'drawing out the sickness' and 'restoring the lost essence'. Most treatments for folk illnesses can be found within cultural references. The Malasars treatment involves praying, communication with the evil sprit and the use of plants.

The Malasars traditional plant classification and nomenclature is complex and unique. During our ethnobotanical survey of the Malasars in the Velliangiri hills, we recorded the food and medicinal use of several ethnotaxa of *Diplocyclos palmatus *(L.) C. Jeffrey. In Tamil it is known as *Iverali*, which is based on it's morphology, meaning palm like leaves (I' = five; veralli = five fingers like leaf). The ethnotaxa were collected from different habitats of which some have utility as either medicine or healthy food. One of the ethnotaxa of *Diplocyclos palmatus *was identified by a Malasars healer as '*Lingankatti'*, which is only used for rheumatic pain. The name *Lingankatti *is derived from morphology of the fruit, which is deep reddish in colour, but more importantly this is also related to spiritual folk lore; *Siva*, the fire God of the holy hills represents red hot volcanic lava. Ragupathy [100], during his ethnobotancial survey of the Irulas in the Coromandal Coast of Thanjavur district of Tamil Nadu, recorded the food and medicinal uses of several ethnotaxa of *Cardiospermum halicacabum*. Some ethnotaxa of *Cardiospermum halicacabum *collected from different places are used as food, while others are used as medicine [101]. An understanding of the multi-mechanistic aboriginal classification may lead to the discovery of new ethnotaxa, which offer novel medicinal and nutritional value [65,25,13].

### Retention of TAK

**Figure 7:**
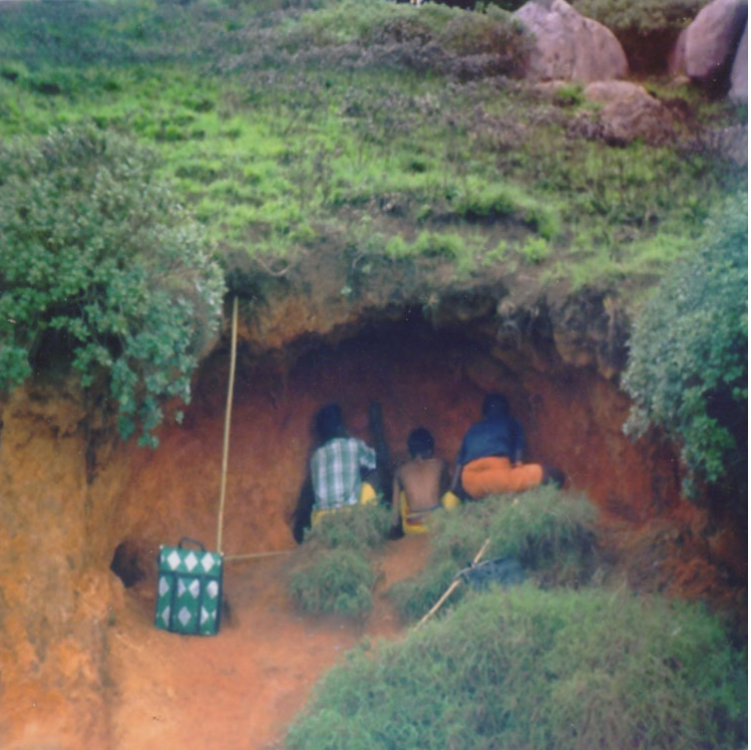
Collection of holy soil and medicinal tubers by pilgrims of 'Thiruneer malai' (1500 msl).

Some of the Malasars TAK of medicinal plants is being passed on to the pilgrims who visit the holy hills. We surveyed pilgrims whom travelled to the Velliangiri hills for medicine and good health. The surveys represented a reasonable understanding of the Malasars' TAK of medicinal plants. Of the 240 pilgrims surveyed 80% could answer all the questions correctly, name and list the medicinal uses of many plants. 60% of the people said they gained their knowledge of plants from the local Malasars healers, 22% of the people said they learned from their parents and 18% of the people had learnt from their fellow pilgrims. The Malasars have helped the pilgrims for many years to navigate the hills and seek medicine and health. Traditionally, knowledge of medicinal plant remedies has been passed from the Malasars to the pilgrims. More recently, some of the modern pilgrims who do not depend on the Malasars for knowledge and guidance through the hills may be causing considerable damage to the ecosystem. During the temple festival season several thousand of these people visit the temple. Government authorities build roads and temporary infrastructure (shops, camping facilities) that disturb the native ecosystems and traditional cultures (Figure 7). There are reports of over collection of plants and the use of firecrackers to keep away wild animals [64]. Conservation of this area is needed to protect the ecosystem, which includes its native people and their knowledge.

The results of this study have demonstrated that medicinal knowledge of plants in the Velliangiri hill, Nilgiri Biosphere Reserve is a well preserved tradition held by the Malasars. There are two other aboriginal groups who live near the Velliangiri hills, namely, the Muthuvans and Irulas. They also have accumulated extensive ethnobotanical knowledge by their long association with the diversity of plants in the Velliangiri hills. Further research is needed to evaluate the consensus of medicinal utility of plants within and among these cultures. This may provide further evidence for culturally specific classifications, utility of plants and the evolution of local ethnotaxa (genetic haplotypes) that offer medicinal or other utility for different cultures. Recent research is investigating the complex mechanisms of aboriginal classifications [65,25]. The application of this research may bridge ancient traditional knowledge with modern molecular tools such as DNA barcoding [102,103] in order to reliably identify new sources of medicines, agricultural cultivars or conservation strategies that have broad implications to society-at-large.

## Conclusion

This article primarily focused on the TAK of the Malasars concerning medicinal flora of the Velliangiri holy hills. We have documented relatively high consensus among the Malasars informants concerning TAK of medicinal plants. The Malasars' healthy lifestyle is supported by the daily intake of plants as part of their diet to maintain good health. There are a few exceptions to diseases found amongst the Malasars in which they are dependent on modern medicine, like vaccinations for polio, small pox and treatment for tuberculosis, which is provided by the government intervention. There are considerable economic benefits in the sharing of this rich TAK with society-at-large. We suggest that TAK health practices should be considered to augment modern primary health care systems. Unfortunately, the Malasars' TAK is limited to local aboriginal communities with some extensions to rural mainstream people who depend on Malasars TAK to sustain their healthy lifestyle. Barriers to the effective dissemination of the Malasars' TAK is likely due to the inferior means of communication, poverty, influence of the modern health care facilities and migration of aboriginals. The rich TAK of the Malasars may be in peril or may even become extinct because of migration. Thousands of pilgrims migrate to the Velliangiri holy hills causing environmental degradation, threatening the native flora, which is the source of the Malasars' medicine. The migration of the younger generation of Malasars from their communities and TAK further threatens the existence of this precious knowledge. We have documented some the Malasars TAK here in order to protect it within our aboriginal repository of knowledge (ARK) research program. This research sheds some light on a traditional culture that believes that a healthy lifestyle is founded on a healthy environment and we suggest that TAK, such as that of the Malasars, may serve toward a global lifestyle of health and environmental sustainability for society-at-large.

## Abbreviations

F_IC_: Factor of Informant Consensus; n_ur_: number of use-reports in each category; n_t_: number of taxa in each category; TAK: Traditional Aboriginal Knowledge; TEK: Traditional Ecological Knowledge; LEK: Local Ecological Knowledge

## Competing interests

The author(s) declare that they have no competing interests.

## Supplementary Material

Additional file 1 Malasars medicinal utility of the flora in the Velliangiri hills. The data provided represent medicinal plant's botanical name, voucher number, Malasars' name, mode of preparation and medicinal use – first hand information gathered from the Malasars aboriginal community. Click here for file
