# Supplementary material for: Consensus of the 'Malasars' traditional aboriginal knowledge of medicinal plants in the Velliangiri holy hills, India
Source: J Ethnobiol Ethnomed. 2008 Mar 27;4:8. doi: 10.1186/1746-4269-4-8 (PMC2323365; doi:10.1186/1746-4269-4-8)
Supplement: Additional file 1 — Malasars medicinal utility of the flora in the Velliangiri hills. The data provided represent medicinal plant's botanical name, voucher number, Malasars' name, mode of preparation and medicinal use – first hand information gathered from the Malasars aboriginal community. [file 1746-4269-4-8-S1.pdf]

Additional file 1. Malasars medicinal utility of the flora in the Velliangiri hills.

Botanical Name and Family	Tamil Lexicon	Method of preparation and medicinal uses
**Acanthaceae**		
Blepharis repens (Vahl) Roth (MM492)	Elumbotti	Leaf juice boiled with sesame oil and applied externally to wounds.
Hygrophila schulli (Schum.-Heiner) M.R. & S.M.A. (MM188)	Neermulli	Leaf decoction used as a diuretic.
Indoneesiella echioides (L.) Sreem. (MM796)	Adukkuchatti	Leaf paste mixed with coconut oil and applied on grey hair for blackening.
Justicia diffusa L. (MM369)	Kodasoori	Leaf paste with sesame oil is used as a remedy for tooth decay.
Justicia tranquebariensis L.f. (MM568)	Punnaku poodu	Root paste applied for tooth ache.
**Aizoaceae**		
Glinus lotoides L. (MM736)	Siruseruppadai	Leaf juice with garlic used as purgative and to cure eczema.
Mollugo nudicaulis Lam. (MM1316)	Parpadagam	Whole plant decoction administered orally for fever.
Trianthema decandra L. (MM487)	Vellai chaarani	Root bark powder taken as Kayakalpa to strengthen the muscles.
**Amaranthaceae**		
Achyranthes aspera L. (MM1617)	Nayuruvi	Leaf juice mixed with pepper and turmeric, and boiled in castor oil. Oil is applied to cuts and wounds.
Amaranthus spinosus L. (MM246)	Mullukeerai	Whole plant ash mixed with coconut oil and used as antiseptic.
**Anacardiaceae**		
Mangifera indica L. (MM963)	Mamaram	Cotyledons of *Mangifera indica* and fruit wall of *Punica granatum* were ground with leaf extracts of *Solanum nigrum* and is used to cure dysentery and diarrhœa.
**Apiaceae**		
Centella asiatica (L.) Urban (MM1698)	Vallarai	Leaf paste used to cure eczema and leucorrhœa.
**Apocynaceae**		
Wrightia tinctoria (Roxb.) R.Br. (MM1257)	Veppalai	Latex and leaf paste applied externally to cure blisters.
Carissa carandas L. (MM679)	Kila	Fruits are eaten when ripe and used for making pickles.
**Araceae**		
Colocasia esculenta (L.) Schott (MM384)	Sembu	Tender leaves and petioles used as greens.
**Arecaceae**		
Phoenix loureiroi Kunth (MM949)	Eechai	Fruits edible.
**Asclepiadaceae**		
Gymnema sylvestre (Retz.) R. Br. Ex Roem. & Schultes. (MM728)	Sirukurinjan	Air dried and powdered leaves mixed with water and given orally for scorpion and rat bites.
Holostemma ada-kodien Schultes (MM1189)	Paalai	Latex is applied on healing blister.

Wattakaka volubilis (L.f.) Stapf. (MM1336)	Perunkurinchan	Leaves ground with pepper and administered orally for dyspepsia.
**Asteraceae**		
Anaphalis lawii (Hook.f.) Gamble (MM763)	Kalthamarai	Whole plant air-dried, powdered, and consumed with food as 'Kayakalpa'.
Eclipta prostrata (L.) L. (MM955)	Karisalankanni	Leaf extract and fruit juice of *Phyllanthus emblica* boiled in coconut oil and applied to grey hair. Leaves used as greens for general health purposes.
Sphaeranthus indicus L. (MM173)	Kottakaranthai	Leaf paste with cumin seeds used to cure dysentery. Whole plant collected before flowering, air dried, powdered, and used to cure skin diseases.
**Begoniaceae**		
Begonia malabarica Lam. (MM332)	Rathachoori	Stem soaked in milk overnight and juice administered orally on empty stomach as Kayakalpa.
**Boraginaceae**		
Coldenia procumbens L. (MM277)	Serupadai	Leaf powder acts as a diuretic.
Heliotropum indicum L. (MM909)	Telkodukku	Leaf juice with sesame oil is used to cure ear ache.
Trichodesma indicum (L.) R.Br. (MM1660)	Kavuthumbai	Leaves are ground with garlic and *Acorus calamus* rhizome, and the paste is applied on wounds.
Cordia monoica Roxb. (MM102)	Pattaimaram	Barks used as alternate to Areca nut for chewing.
**Burseraceae**		
Canarium strictum Roxb. (MM1982)	Kungilium	Resin is grounded with turmeric, made into paste, and applied over heel cracks.
**Combretaceae**		
Terminalia arjuna (DC.) W. & A. (MM1132)	Marutham	Bark juice administered orally to cure heart diseases.
Terminalia bellirica (Gaertner) Roxb. (MM689)	Thanikkai	Fruit juice with buttermilk administered orally for stomach ache.
Terminalia chebula Retz. (MM410)	Kadukkai	Fruit juice mixed with honey taken orally to cure coughing.
**Convolvulaceae**		
Evolvulus alsinoides L. (MM1144)	Vishnukiranthi	Whole plant powder taken as a 'Kayakalpa' to improve memory.
Ipomoea obscura (L.) Ker Gawler. (MM807)	Siruoonan	Warmed leaves ground with castor oil and applied on blisters.
**Crassulaceae**		
Kalanchoe floribunda W. & A. (MM715)	Ranakalli	Leaf juice administered orally to cure leucoderma and skin diseases.
**Cucurbitaceae**		
Coccinea grandis (L.) J. Voigt (MM495)	Kovai	Whole plant extract used as diuretic.
Diplocyclos palmatus (L.) C. Jeffrey (MM555A)	Iverali	Leaves dried/evoked and eaten.
Diplocyclos palmatus (L.) C. Jeffrey (MM555)	Lingankatti	Leaf paste applied externally to reduce rheumatic pain.
Kedrostis foetidissima (Jacq.) Cogn. (MM376)	Appakovai	Leaf juice applied externally on joints to cure diarrhœa in babies 3-4 month old.

Mukia maderaspatana (L.) M.Roem (MM1219)	Mumusukai	Leaf juice administered orally to cure bronchitis.
**Euphorbiaceae**		
Acalypha fruticosa Forssk. (MM984)	Sirusinni	Fresh leaves are eaten with salt to get relief from stomach ache. Leaves used as greens.
Acalypha indica L. (MM1454)	Kuppaimeni	Leaf paste applied externally for skin allergies.
Euphorbia hirta L. (MM48)	Ammanpacharisi	Latex applied externally to cure wounds.
Euphorbia thymifolia L. (MM848)	Siruamman pacharisi	Leaves crushed with butter milk and administered orally for jaundice.
Flueggea leucopyrus Willd. (MM1481)	Veppolan	Leaves crushed with butter milk and administered orally for leucorrhœa.
Phyllanthus emblica L. (MM842)	Nellikai	Fruit decoction is given for blood pressure.
**Fabaceae**		
Abrus precatorius L. (MM1652)	Karunkutri	Leaf ash mixed with coconut water and applied to forehead to keep away evil spirits.
Abrus precatorius L. (MM1651A)	Kurunkundumani	Fresh leaves are chewed to prevent dental disorders.
Clitoria ternatea L. (MM986)	Sangupushpam	Flowers used to worship God-Lord Siva.
Crotalaria verrucosa L. (MM1462)	Kilukiluppai	Root tied around waist to keep away evil spirits.
Indigofera aspalathoides Vahl ex DC. (MM1194)	Sivanar vembu	Root paste brushed on decayed, aching teeth.
Indigofera caerulea Roxb. (MM25)	Avuri	Leaf juice administered orally to cure night blindness. Root juice is given for jaundice and epilepsy.
Indigofera parviflora Heyne ex W.& A. (MM1003)	Neeli	Mixed with a leaf paste and applied to scorpion bites.
**Laminaceae**		
Leucas aspera (Willd.) Link. (MM727)	Thumbai	Leaf juice mixed with honey and administered orally for bronchitis. Tender leaves rolled with salt are kept in mouth to cure teeth ache and gums swelling. Flowers used for spiritual-offering to God.
**Lauraceae**		
Cinnamomum macrocarpum Hook.f. (MM1551)	Lavangam	Bark decoction administered orally for cough.
**Lycopodiaceae**		
Lycopodium phlegmaria L. (MM08)	Sivanjadai	Plant paste applied over the body before bath to prevent children from obtaining skin diseases.
**Lythraceae**		
Ammania baccifera L. (MM713)	Kalluruvi	Leaf paste applied externally to cure muscular pains.
**Malvaceae**		
Pavonia procumbens (W. & A.) Walp. (MM382)	Palampasi	Leaves ground with onion bulb, cumin seeds, and administered orally along with buttermilk to cure peptic ulcers.

Sida rhombifolia L.. (MM997)	Kuruthoti	Whole plant paste applied on forehead to cure headache.
**Meliaceae**		
Azadirachta indica Adr. Juss. (MM1497)	Veppamaram	Leaf decoction is used for ulcers, eczema and as antiseptic.
**Menispermaceae**		
Cocculus hirsutus (L.) Diels. (MM1609)	Jalanthirati	Leaf juice mixed with equal amount of water to form gel-like substance, which is administered orally to reduce body temperature.
Tinospora cordifolia (Willd.) Miers ex Hook.f. & Thoms. (MM168)	Chintil	Leaf juice along with buttermilk administered orally to cure piles.
**Mimosaceae**		
Dichrostachys cinerea (L.) W. & A. (MM863)	Vedathalai	Leaves eaten with ragi pancake and jaggery to cure leucorrhœa.
Mimosa pudica L. (MM864)	Thottal sinungi	Leaves boiled with sesame oil and applied externally for psoriasis.
**Myristicaceae**		
Myristica dactyloides Gaertner (MM850)	Jathikkai	Seed paste with water administered orally to cure dysentery.
**Nyctaginaceae**		
Boerhaavia diffusa L. (MM588)	Kattu saranai	Root decoction is given as a remedy for arthritis.
Boerhavia erecta L. (MM276)	Saranai	Whole plant juice administered orally to remove stones from urinary tracts.
**Orchidaceae**		
Bulbophyllum fusco-purpureum Wight. (MM1152)	Throbathi manjal	Juice of pseudobulb is taken orally to reduce body temperature.
Coelogyne nervosa A. Rich. (MM1142)	Seethaimanjal	Leaf paste applied on the body of children, followed by bathing to maintain good health.
Malaxis rheedii Sw. (MM1356)	Kattuvengayam	Whole plant paste applied externally to cure blisters and wounds.
**Oxalidaceae**		
Oxalis corniculata L. (MM1169)	Aarai	Leaves used as greens.
**Papaveraceae**		
Argemone mexicana L. (MM675)	Pirama thandu	Latex is applied on blisters.
**Periplocaceae**		
Hemidesmus indicus (L.) R.Br. (MM617)	Nannari	Root extract is used as a stimulant to increase blood circulation.
**Piperaceae**		
Peperomia wightiana Miq. (MM47)	Kalporumi	Leaf juice administered orally to cure gastric disorders.
Piper longum L. (MM854)	Thipili	Ash of the seed taken orally with honey to cure cough.
**Plumbaginaceae**		
Plumbago zeylanica L. (MM821)	Kodiveli	Root bark powdered and administered orally with jaggery to cure leucorrhœa.
**Pteridaceae**		
Pteridium aquilinum (L.) Kuhn (MM768A)	Surulkeerai	Leaves used as greens.
**Rhamnaceae**		
Zizyphus mauritiana Lam. (MM1455)	Elanthai	Leaf decoction is administered orally to cure piles. Fruits edible as snack.

Zizyphus oenoplia (L.) Mill. (MM1605)	Soori	Fruits edible.
Scutia myrtina (Burm.f.) Kurz. (MM242)	Karunchoori	Fruits edible.
**Rosaceae**		
Rubus niveus Thunb. (MM333)	Semmullu	Fruits edible.
**Rubiaciae**		
Oldenlandia umbellata L. (MM638)	Saayaver	Root paste taken orally along with garlic to remove intestinal worms.
**Rutaceae**		
Aegle marmelos (L.) Corr. (MM822)	Vilvam	Leaves ground with pepper and milk and administered orally to cure dyspepsia.
Limonia acidissima L. (MM191)	Vilamaram	Leaf decoction with cumin seeds is taken for giddiness. Fruits edible.
Naringi crenulata (Roxb.) Nicolson (MM719)	Mahavilvam	Leaf paste administered orally along with milk to cure mental disorders.
Toddalia asiatica (L.) Lam. (MM189)	Milakaranai	Root bark powdered and used to cure stomach ulcers. Leaves used as greens for general health
**Salvadoraceae**		
Azima tetracantha Lam. (MM1533)	Sangumullu	Root paste applied externally on wounds.
**Sapindaceae**		
Cardiospermum halicacabum L. (MM247)	Mudakatthan	Whole plant boiled with dried ginger and cumin seeds and the decoction consumed orally to cure rheumatism.
Dodonaea angustifolia L.f. (MM130)	Virali	Leaves boiled with water and used for taking bath to get relief from paralysis
**Scrophulariaceae**		
Scoparia dulcis L. (MM801)	Sakkarai vembu	Whole plant is crushed to powder and administered orally with water to reduce blood pressure.
**Selaginellaceae**		
Selaginella rupestris (L.) Spring (MM47A)	Karudapacchai	Whole plant is tied around waist of children to keep away evil spirits.
**Solanaceae**		
Datura metel L. (MM1512)	Karuoomathai	Leaf and fruit juice boiled with buffalo milk and sesame oil, and is used to cure aches. Dried leaves are rolled and smoked like beedi to get relief from cold.
**Sterculiaceae**		
Helicteres isora L. (MM740)	Valampuri	Fruit boiled with pepper, garlic, and sesame oil and poured into ears to cure ear ache.
**Tiliaceae**		
Grewia hirsuta Vahl. (MM1359)	Kattu kadalai	Fruits edible.
**Verbenaceae**		
Lippia javanica (Burm.f.) Spreng. (MM1903)	Narimiratti	Leaf juice administered orally with pepper powder to cure digestive disorders.
Phyla nodiflora (L.) Greene (MM1715)	Poduthalai	Tender leaf juice is given to children to cure diarrhœa. Also the juice applied to head to cure dandruff.
**Vitaceae**		

Cissus quadrangularis L. (MM1086)	Pirandai	Young tender stem used for making salads.
**Zingiberaceae**		
Acorus calamus Linn. (MM0111)	Vasampu	Rhizome paste mixed with the ash of peacock's feather and honey. Administered orally to cure cough.
